# Processing Cellulose Acetate into Hierarchically Porous Monoliths via Thermally Impacted Nonsolvent-induced Phase Separation with Octanol: Application in Dye Adsorption

**DOI:** 10.1021/acsomega.5c08761

**Published:** 2025-12-22

**Authors:** Poliana Ricci, Brenda F. Silva, Marcos V. Ferreira, Henrique A. Sobreira, Allyson L. R. Santos, Anizio M. Faria, Rosana M. N. Assunção

**Affiliations:** † Institute of Chemistry, 28119 Federal University of Uberlândia, Uberlândia 38400-902, Brazil; ‡ Institute of Exact and Natural Sciences of Pontal, Federal University of Uberlândia, Ituiutaba 38304-402, Brazil; § Federal Institute of Education, Science and Technology of Triângulo Mineiro, Uberaba 38305-200, Brazil

## Abstract

This study reports the fabrication of hierarchically porous cellulose acetate (CA) monoliths via thermally impacted nonsolvent-induced phase separation (TIPS/NIPS), employing *n*-octanol as a novel nonsolvent. A ternary phase diagram (TPD) of the CA/DMF/*n*-octanol system was constructed to define optimal processing conditions in the metastable region, enabling the formation of continuous monolithic structures. The resulting CA monoliths exhibited a sponge-like morphology with interconnected meso- and macropores, a specific surface area of 34.33 m^2^ g^–1^, a high porosity of 90.68 ± 0.66%, and a low density of 103.12 mg cm^–3^. Adsorption studies using crystal violet (CV) as a model contaminant demonstrated efficient dye removal (∼90%) within 180 min, reaching the highest adsorption capacity of 0.92948 mg g^–1^, as determined by the Langmuir model. Kinetic modeling indicated that the adsorption followed a pseudo-second-order mechanism, suggesting chemisorption, whereas the equilibrium data fit best to the Langmuir isotherm model, indicating monolayer adsorption. Desorption studies showed that methanol and ethanol enabled CV recovery with efficiencies of up to 70%. These results confirm that the combination of TIPS/NIPS and *n*-octanol provides a promising, tunable platform for producing sustainable CA-based monoliths for wastewater treatment and dye removal.

## Graphical abstract

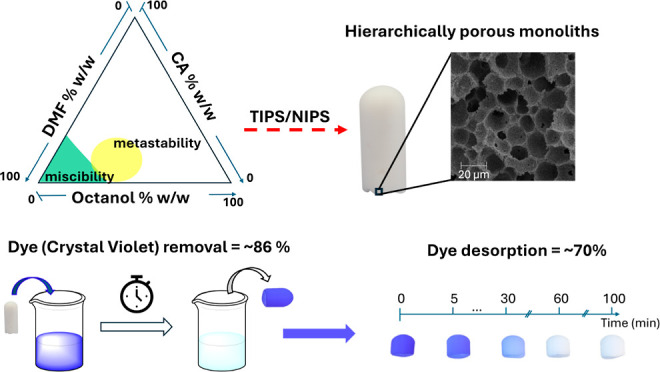

## 1 Introduction

The development of hierarchically porous polymeric monoliths presents exciting opportunities for advanced material design in various fields, including separations, catalysis, sensing, and environmental remediation.^1,2^ Among polymeric monolith materials, cellulose derivatives are particularly appealing due to their sustainability, versatility, and favorable processing characteristics.^3,4^

Cellulose diacetate (CA), one of the most widely used cellulose derivatives, combines the intrinsic advantages of cellulose—biodegradability, biocompatibility, and abundant surface functional groups—with excellent processability in organic solvents and tunable thermal and mechanical properties.^5,6^ These features make CA a promising candidate for fabricating porous monolithic structures. Processing CA into monoliths opens new pathways for applications such as adsorption and separation, where controlled porosity and mass transport are key.

A critical factor in monolith fabrication is the ability to control pore morphology across multiple length scales.^7^ Phase separation techniques, in particular nonsolvent-induced phase separation (NIPS) and thermally impacted nonsolvent-induced phase separation (TIPS/NIPS), enable the creation of hierarchically porous CA structures from solution precursors.^8,9^ In TIPS/NIPS, selecting an appropriate nonsolvent is a crucial factor in driving phase separation and determining the final monolith morphology.^10^

In TIPS/NIPS processes, the competition between thermodynamic and kinetic factors determines the final pore morphology, where solvent–nonsolvent exchange rate and volatility are key parameters.^11^ Compared with short-chain alcohols, which promote rapid demixing and droplet-type morphologies, longer-chain alcohols such as *n*-octanol moderate diffusion and stabilize spinodal decomposition, enabling more interconnected and hierarchically porous networks.^8^

In this study, the controlled fabrication of porous cellulose acetate (CA) monoliths aims to produce sustainable, structurally optimized adsorbents, with the main challenge being directing phase separation to achieve hierarchical porosity that combines macro-, meso-, and micropores. This morphology results from the dynamics of liquid–liquid phase separation during matrix solidification, within the metastable region of the ternary phase diagram. Spinodal decomposition generates continuous structures with interconnected macropores, while subsequent demixing produces meso and micropores that increase surface area and adsorption capacity.^12^

Octanol’s moderate polarity, low volatility, and unique solvent-interaction properties offer an innovative approach to tune phase-separation dynamics and control pore structure, stabilizing spinodal domains and promoting a smooth pore-size transition. To our knowledge, the use of octanol in the fabrication of CA-based monoliths via TIPS/NIPS has not been systematically investigated. This approach offers a promising alternative to conventional nonsolvents (e.g., water, ethanol) and expands the design space for CA monoliths with hierarchical porosity, enhanced mechanical integrity, and efficient mass transport, key features for advanced adsorption and environmental applications.

The porous architecture of polymeric monoliths is highly relevant for applications that utilize adsorption. To demonstrate the practical applicability of the developed CA monoliths, we selected the adsorption of crystal violet (CV) dye as a model case. CV is a persistent, toxic triarylmethane dye widely used in the textile, pharmaceutical, and paper industries. Due to its complex molecular structure and high stability, CV is resistant to biodegradation and poses a serious threat to aquatic ecosystems and human health.^13,14^ The effective removal of CV from wastewater is therefore a crucial environmental remediation challenge.

Beyond demonstrating removal efficiency, a detailed evaluation of the adsorption kinetics and equilibrium behavior is essential for understanding the practical applicability of the developed monoliths. The adsorption rate and equilibrium time are critical factors that govern the selection of suitable adsorbent materials for water treatment. Experimental parameters such as pH, ionic strength, temperature, solute concentration, sorbent dose, and adsorbent texture all influence adsorption kinetics.^15–18^ To this end, the following kinetic models were applied to analyze the adsorption process in this study: the pseudo-first-order, pseudo-second-order, Elovich, and intraparticle diffusion models.^19–22^

In this context, the present work aims to develop hierarchically porous CA monoliths via TIPS/NIPS using octanol as a novel nonsolvent and to evaluate their performance as adsorbents for the removal of CV from aqueous effluents. By combining studies of morphology, adsorption kinetics, and equilibrium isotherms, we provide new insights into the design and application of CA-based monoliths for environmental remediation.

## 2 Materials and Methods

### 2.1 Materials

The aqueous solutions were prepared using ultrapure water (ASTM type I, resistivity ≥18 MΩ cm) produced by a Megapurity system (Billerica, USA). Cellulose acetate (CA) was supplied by Rhodia Solvay (Santo André, Brazil) with a degree of substitution (DS) of 2.28 and in the form of flakes. *N*,*N*-Dimethylformamide (DMF) was purchased from Synth (Diadema, Brazil). Octanol and ethanol were acquired from Dinâmica (Indaiatuba, Brazil), and CV was supplied by Isofar (Rio de Janeiro, Brazil). All reagents were used without further purification.

### 2.2 Building a Ternary Phase Diagram (TPD) for a Cellulose Acetate–DMF–*n*-Octanol System Using the Cloud Point Method at 70 °C

The development of monoliths involved studying the phase diagrams of the polymer/solvent/nonsolvent system using the cloud-point method. In this study, nine solutions of cellulose acetate (CA) in *N*,*N*-dimethylformamide (DMF) with concentrations ranging from 2 to 18 wt % were prepared at room temperature (24 °C ± 2 °C) under magnetic stirring. The phase-separation process was monitored by measuring the mixture’s turbidity (cloud point) during dropwise addition of *n*-octanol at 70 °C. In these experiments, *n*-octanol served both as a nonsolvent to induce phase separation and as a porogenic agent contributing to the hierarchical pore structure.

The experiments were performed at 70 °C to investigate the influence of the thermally impacted nonsolvent-induced phase separation (TIPS/NIPS) on TPD. The titration process was concluded when permanent turbidity was observed in the system, and the volume of the titrant (*n*-octanol) used was recorded.

### 2.3 Preparation of the CA Monoliths

Based on the TPD results, the conditions for producing CA monoliths were established as follows: a 17.5 wt % CA solution in DMF was prepared in a thermostatic bath at 70 °C, and *n*-octanol was added dropwise at a solvent/nonsolvent ratio of 1:1 (v/v). The amount of *n*-octanol added was sufficient to induce turbidity in the solution without causing effective solid–liquid phase separation. The mixture was then transferred to glass test tubes, cooled to approximately 24 °C, and left undisturbed at room temperature (24 °C) for 7 to 15 days to allow for slow phase separation and gel formation.

After gel formation, the solvent-exchange process was performed to preserve the monolith structure. This step was initiated only after confirming complete phase separation, thereby preventing ethanol from altering the gel. Ethanol was used solely to remove DMF and the nonsolvent. Initially, ethanol was added to fully immerse the gel in each test tube, facilitating the removal of the monolith from the tube. The monoliths were subsequently submerged in fresh ethanol, washed three times with different aliquots, and finally transferred to ultrapure water to completely remove residual solvents and nonsolvents. The resulting CA monoliths were stored in sample containers with a methanol–water mixture (1:1) until further use.

### 2.4 Characterization of CA Monoliths

Fourier-transform infrared (FT-IR) spectroscopy measurements were performed using the attenuated total reflectance (ATR) method with an Agilent Technologies Cary 630 FTIR spectrometer (Santa Clara, CA, USA) equipped with an ATR (Smart Orbit) accessory. Nitrogen adsorption/desorption isotherms were measured using an automatic physisorption analyzer (Micromeritics ASAP 2020 Plus, Norcross, GA, USA). The specific surface area (SSA) was determined from nitrogen adsorption–desorption isotherms at −195.5 °C using the Brunauer–Emmett–Teller (BET) method. Pore size and distribution were characterized using the progressive pore-emptying theory, which involves decreasing pressure. Scanning electron microscopy (SEM) images were recorded using a VEGA3 TESCAN scanning electron microscope (Brno, Czech Republic) operating at 10 kV. A thin gold film was sputtered on the samples before the images were collected using a Quorum QR 150ES Metalizer for carbon and gold. X-ray diffraction (XRD) spectra were obtained on a Shimadzu XRD-6000 at a scanning rate of 4.0° min^–1^ from 5 to 40° (2θ) at 40 kV and 30 mA using CuKα radiation. The degree of crystallinity was calculated using a deconvolution method that separated crystalline peaks from the amorphous halo in the diffractograms. The thermogravimetric curves (TGA) were obtained using a Discovery thermogravimetric analyzer (TGA 55, TA Instruments, New Castle, DE, USA) from room temperature to 600 °C at a heating rate of 10 °C min^–1^ under a nitrogen atmosphere with a flow rate of 50 cm^3^ min^–1^. Differential scanning calorimetry (DSC) experiments were conducted using a Differential Scanning Calorimeter model Discovery DSC25 (TA Instruments, New Castle, DE, USA). Approximately 3 mg of the samples was heated from −90 to 300 °C to record the first thermal scan. The equipment was cooled by a Refrigerated Cooling System (RCS120) to −90 °C, then heated to 260 °C to record the second scan of thermal events. The heating rate used was 10 °C min^–1^ in a nitrogen atmosphere with a flow rate of 50 cm^3^ min^–1^. UV–vis absorbance measurements were carried out using a PerkinElmer LAMBDA 25 UV–vis spectrometer.

### 2.5 Determination of Monolith Macroporosity and Density

The monolith’s porosity (*P* %) was evaluated using the methodology described by Raja et al. (2014) and Gou et al. (2021).^23,24^ In this method, preweighed, dried monoliths were immersed in *n*-butanol for 2 h. Afterward, the monoliths were gently dried with absorbent paper to remove excess solvent, then weighed. The macroporosity was calculated using eq 1.

1 $$P\;\%=\frac{\left(\frac{M_{1}}{\rho_{1}}\right)}{\left(\frac{M_{1}}{\rho_{1}}+\frac{M_{2}}{\rho_{2}}\right)}\times 100\%$$

where *M*_2_ is the mass of the monolith after immersion in *n*-butanol, *M*_1_ is the mass of the dry monolith, ρ_1_ is the density of *n*-butanol, and ρ_2_ is the density of the pure polymer.

Their weight and volume determined the density (ρCA) of the CA monoliths. The volume was calculated considering the monolith’s length and diameter.

### 2.6 Application of CA Monoliths in Dye Removal Studies

#### 2.6.1 Kinetic Study of Crystal Violet (CV) Removal

The adsorption/desorption capacity of the CA monoliths was evaluated using crystal violet (CV), a violet-colored ionic dye with a UV–vis absorption peak at 590 nm (Figure S1a). To quantify CV concentrations, a standard curve was built in distilled water (0.5–5.6 mg L^–1^, Figure S1b) using UV–vis spectroscopy.

Kinetic adsorption experiments were conducted in triplicate at 22.0 ± 1.5 °C. A 10 mL CV (pH 6.0) solution was stirred with monoliths with dry mass ranging from 0.070 to 0.093 g. CV concentrations varied from 0.5 to 5.6 mg L^–1^. At predetermined intervals 2.5. mL aliquots were withdrawn, analyzed spectrophotometrically, and returned to the beaker. The analyses continued until the absorbance intensities were steady, typically after 180 min. All experiments were performed in triplicate.

The removal percentage (*R* %) and adsorbed amount (*q*_*t*_) were calculated using eqs 2 and 3.

2 $$R\;\left(\%\right)=\frac{\left(C_{0}-C_{t}\right)}{C_{0}}\times 100$$

3 $$q_{t}=\frac{\left(C_{0}-C_{t}\right)}{m}\times V$$

where *C*_0_ and *C*_*t*_ are the initial and time-dependent CV concentrations (mg L^–1^), *m* is the monolith mass (g), and *V* is the solution volume (L).

#### 2.6.2 Adsorption Isotherm Study

Isotherm studies were performed with CV concentrations ranging from 0.5 to 50 mg L^–1^, maintaining a 10 mL solution with 0.070–0.093 g of monolith at 22 °C and pH 6. Aliquots (2.5 mL) were analyzed spectrophotometrically to determine the equilibrium adsorption capacity (*q*_eq_), using eq 4.

4 $$q_{eq}=\frac{\left(C_{0}-C_{eq}\right)}{m}\times V$$

where *C*_0_ and *C*_eq_ are the initial and equilibrium CV concentrations, respectively, and *m* is the monolith mass (g).

#### 2.6.3 Desorption Study

Experiments were carried out using methanol, ethanol, and aqueous solutions with varying pH levels (specifically pH 2.0 and pH 9.0) as extractant solvents. Presaturated monoliths, weighing 0.930–1.460 g, were stirred in 10 mL of the extractant solvent for 100 min. Duplicate experiments were conducted to ensure the reproducibility of the results. The quantities of desorbed CV (*q*_des_) and desorption efficiency (*E*_des_) were calculated using eqs 5 and 6

5 $$q_{des}=\frac{C_{d}\cdot V_{es}}{m_{s}}$$

6 $$E_{des}=\left(\frac{q_{des}}{q_{eq}}\right)\times 100$$

where *C*_d_ is the CV concentration in the extracting solution (mg L^–1^), *V*_es_ is the volume of extractant solvent, and *m*_s_ is the mass of the saturated monolith (mg g^–1^).

## 3 Results and Discussion

### 3.1 Development and Characterization of CA Monoliths

The selection of solvents and nonsolvents is pivotal in the processing methodologies used to fabricate monoliths. Cellulose acetate (CA) exhibits solubility in various solvents, including acetone, dichloromethane, tetrahydrofuran, *N*,*N*-dimethylacetamide, and *N*,*N*-dimethylformamide (DMF). Nonetheless, the solvents acetone, dichloromethane, and tetrahydrofuran exhibit low boiling points and high volatility, which present challenges when implementing thermally induced nonsolvent phase separation (TIPS/NIPS) techniques. The rapid evaporation of high-volatility solvents can result in inadequate control over phase separation and subsequent morphological characteristics. Consequently, DMF is favored due to its elevated boiling point of 153 °C and relatively low volatility, characterized by a vapor pressure of 0.439 kPa at 25 °C.^25^ These properties render DMF particularly suitable for TIPS/NIPS applications conducted at 70 °C, facilitating better control over the resulting structure.

The selection of a nonsolvent significantly affects the morphology during the preparation of porous polymeric materials. In this research, *n*-octanol was chosen as the nonsolvent for the fabrication of cellulose acetate (CA) monoliths due to its compatibility with dimethylformamide (DMF), inert behavior to CA, its high boiling point (195.1 °C), and its low vapor pressure (0.01 kPa at 25 °C), all of which render it suitable for the TIPS/NIPS technique.^25^

To optimize the processing of cellulose acetate (CA) monoliths, ternary phase diagrams (TPDs) were developed using CA, *N*,*N*-dimethylformamide (DMF), and *n*-octanol as a nonsolvent in this investigation. This study focused on the mechanisms of thermally induced nonsolvent phase separation (TIPS/NIPS).

#### 3.1.1 Construction of Ternary Phase Diagrams (TPDs)

Figure 1 illustrates the ternary phase diagram (TPD) for the CA/DMF/nonsolvent system. This diagram offers essential insights into identifying appropriate regions for monolith production and developing porous structures. It defines equilibrium, miscibility, and immiscibility, as well as the binodal curve that separates single-phase regions from liquid–liquid phase-separated regions.

**Figure 1:**
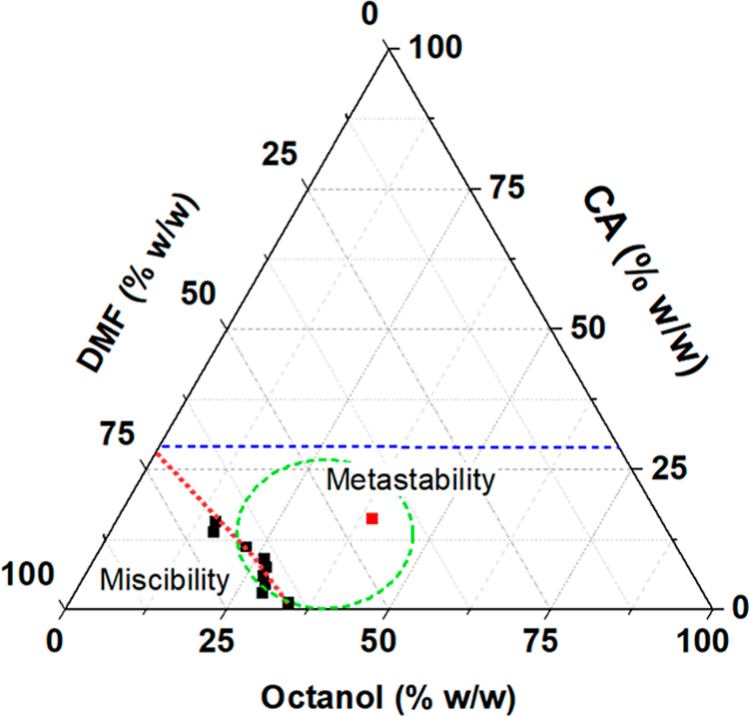
Ternary phase diagram of the CA/DMF/*n*-octanol system at 24 °C. CA concentration ranging from 0.5 to 18 wt %, and the nonsolvent *n*-octanol was gradually added until turbidity was observed, with values typically ranging from 13 to 37 wt %.

The analysis of the CA/DMF/*n*-octanol system (Figure 1) reveals a binodal curve separating the miscible and immiscible regions. We identified the single-phase region, the equilibrium area near the binodal curve, and the two-phase region. Experimental observations suggest that the position of the binodal curve may change due to metastability in its vicinity.^26^

The solvent-to-nonsolvent ratio influences the miscibility region. For the CA/DMF/*n*-octanol system, the binodal curve spans 13–37 wt % *n*-octanol as the DMF concentration increases from 70 to 95 wt %. As a consequence of changes in DMF concentration, the CA concentration increases from 0.5 to 18 wt %, and smaller amounts of *n*-octanol are required to approach the miscibility limits.

It is essential to note that while increasing the polymer concentration can enhance the definition of the binodal curve, this is limited by the solubility of CA in DMF, which allows concentrations of approximately 30 wt %. Additionally, due to the experimental limitations of the cloud point method, the minimum concentration for CA/DMF solutions was set at 18 wt %.

Therefore, based on the TPD diagram, we selected a point in the metastability region (the square point in the red-circled area in Figure 1), corresponding to 17.5 wt %.% CA, 42.1 wt % octanol, and 47.8 wt % DMF (or 1.135:1 w/w, and 1:1 v/v of DMF/octanol) as the conditions to form the CA monoliths. By utilizing this point in the metastable region, we ensured CA miscibility upon heating to 70 °C and a thermal-induced phase transition upon cooling to room temperature (24 °C), resulting in the formation of the gel phase.

#### 3.1.2 Preparation of CA Based on the Ternary Phase Diagram

The procedures involved in forming the monolith are illustrated in Figure 2. The optimal DMF/*n*-octanol ratio for phase separation in the CA system (17.5 wt % in DMF) was 1.0/1.0 (v/v) or 1.136/1.0 (wt %) DMF/octanol, as determined by the TPD (Figure 1).

**Figure 2:**
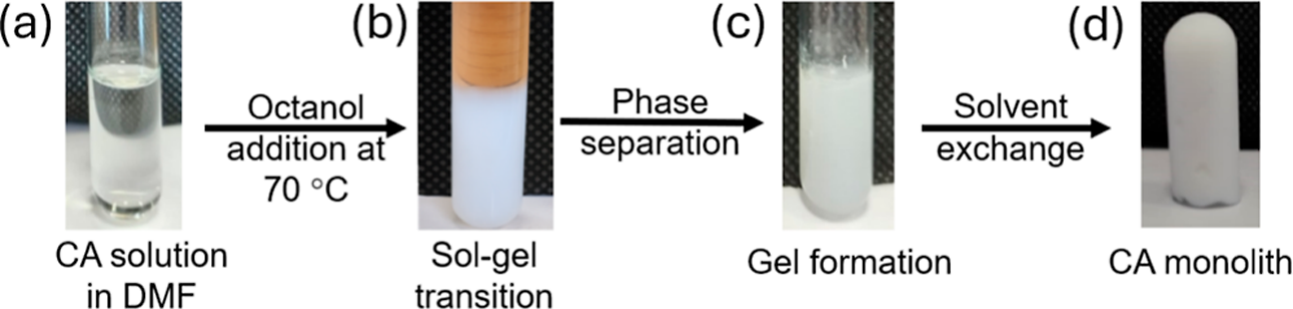
Schematic flowchart of the CA monolith preparation using thermally impacted nonsolvent-induced phase separation (TIPS/NIPS). (a) CA solution (17.5 wt %) in DMF at 70 °C; (b) addition of *n*-octanol to induce phase separation (DMF/*n*-octanol ratio of 1.136/1.0 wt %); (c) CA monolith obtained after washing with ethanol and water gelation during cooling to room temperature (24 °C); and (d) CA monolith obtained after solvent exchange with ethanol and rinsing with water.

Figure 2b illustrates the clouding of the CA/DMF solution upon adding the *n*-octanol at 70 °C. This phase separation is induced by the addition of a nonsolvent and by a reduction in temperature from 70 to 25 °C, which is maintained until the monolithic structure forms. This structure formation typically took 7 to 15 days, as the solvent progressively evaporated (Figure 2c), leading to the development of a monolith in the shape of the container (Figure 2d).

During nonsolvent addition, two types of phase separation may occur: liquid–liquid and solid–liquid.^27^ Solid–liquid phase separation is unsuitable for monolith production because it leads to immediate polymer precipitation, hindering the formation of a porous morphology. In contrast, liquid–liquid phase separation produces a turbid system without direct polymer precipitation. This process results in the formation of a porous three-dimensional interpenetrating network composed of a polymer-rich phase (polymer/solvent) and a polymer-poor phase (nonsolvent and residual solvent).^26^

#### 3.1.3 Characterization of the CA Polymer and CA Monoliths

FTIR spectroscopy was used to investigate the functional groups in the cellulose acetate (CA) monoliths and to confirm the removal of any residual solvents. The resulting FTIR spectra (Figure S2), highlight key assignments at the following wavenumbers: 3475 cm^–1^ (indicative of νO–H groups from the CA polymer and water adsorbed), 2922 cm^–1^ and 2886 cm^–1^ (related to the ν_symmetric_ and ν_asymmetric_ C–H bonds in CH_3_ and CH_2_ groups), 1736 cm^–1^ (corresponding to νC=O of carbonyl ester), 1222 cm^–1^ (associated with νC–C–O), 1032 cm^–1^ (reflecting νC–O), and 899 cm^–1^ (of the νC1–O–C4 glycosidic bond).^28^ Therefore, the CA absorption bands were clearly discernible in the spectra of the monoliths, with no significant changes in intensity, peak shifts, or the appearance of additional spectral features. This observation strongly confirms that the polymer matrix’s structural integrity was preserved during the entire monolith synthesis process. Additionally, the absence of detectable residual solvents or nonsolvent residues was confirmed, corroborating the complete removal of these constituents during monolith fabrication.

Thermogravimetric analysis (TGA) and differential scanning calorimetry (DSC) were employed to assess the thermal behavior of the unprocessed CA polymer and monoliths and to further investigate the presence of residual solvents from processing (Figure 3).

**Figure 3:**
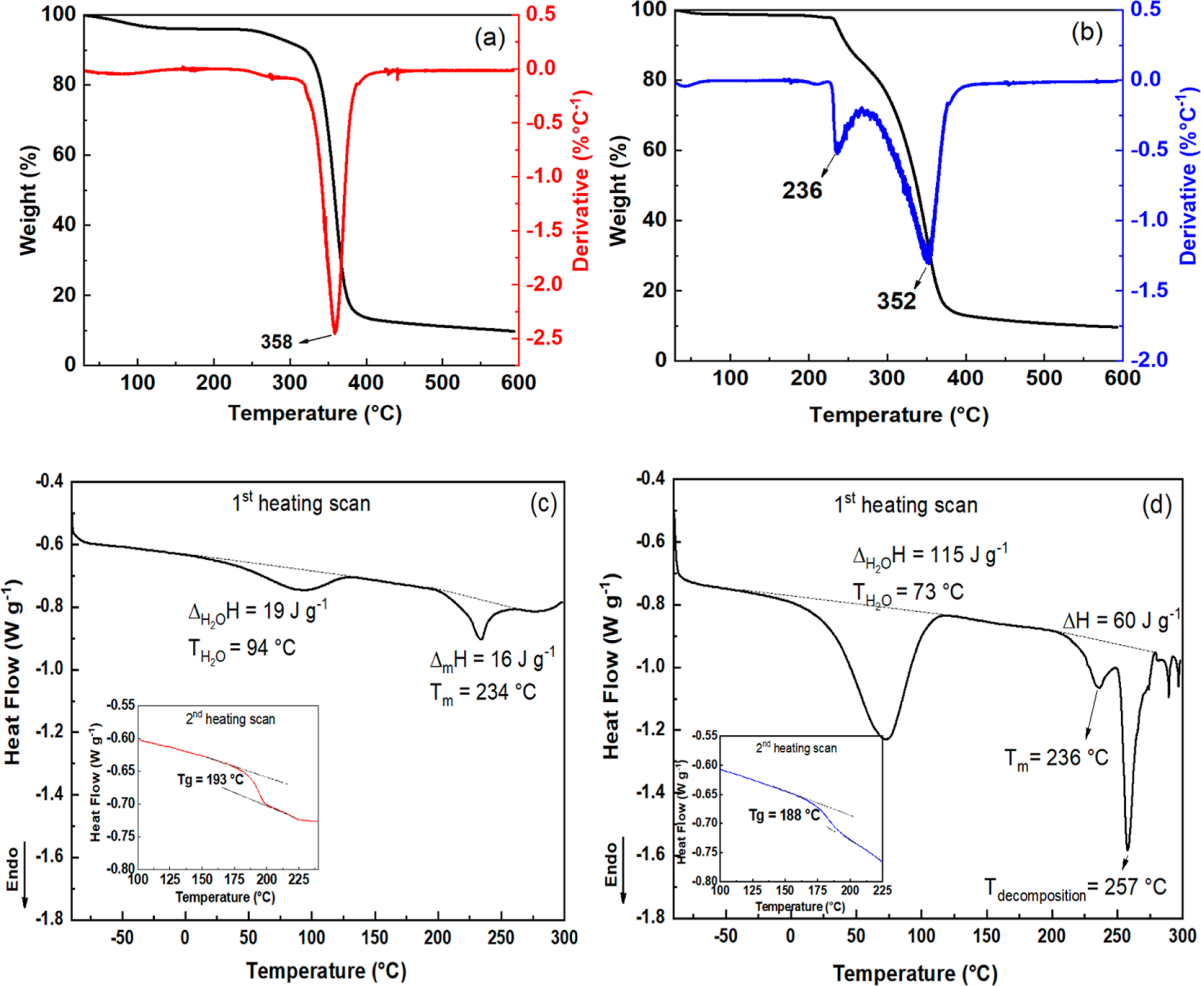
TGA and DTG curves for (a) pristine CA and (b) CA monolith. DSC curves from the first and second heating scans for (c) pristine CA and (d) CA monolith. Analyses were performed under an N_2_ atmosphere (50 mL min^–1^) with a heating rate of 10 °C min^–1^.

The TGA curve of CA (Figure 3a) reveals three main events: (i) between 25 and 200 °C, corresponding to water desorption and volatile compounds evaporation (∼3.9% mass loss); (ii) between ∼200 and 380 °C, due to acetyl group removal (deacetylation) and main chain decomposition; and (iii) above 380 °C, related to carbonaceous material degradation, leaving ∼10% residue at 600 °C.^29,30^ Onset temperatures (*T*_onset_) for these events are summarized in Table S1 and calculated as the intersection of the initial baseline and the tangent to the maximum gradient of the TGA curve.

Similar thermal events were observed for the monoliths, with variations in the mass–loss profiles (Figure 3b). The first event (below 100 °C) is attributed to the removal of surface-adsorbed water, corresponding to approximately 1.4% weight loss. The second event, with a maximum decomposition (*T*_max_) of 352 °C, is slightly lower than that of CA (358 °C). The third event, which begins after the extrapolated end point of the second event (*T*_endset_), involves polymer chain decomposition and carbonization, with residue percentages of nearly 10%. The CA monolith exhibited an additional thermal event between the first and second CA events, with a *T*_max_ of 236 °C, which may be related to morphological changes induced by the porous structures, making the monolith surface more susceptible to heat and decomposition. These differences likely reflect morphological changes during monolith processing.

DSC analysis of CA (Figure 3c) for the first heating scan reveals two endothermic events: (i) occurring between 0 and 135 °C, which is associated with water desorption, and (ii) between 189 and 265 °C, which corresponds to the melting of CA, with a peak at 234 °C and a melting temperature (Δ_m_ *H*) of 16 J g^–1^, followed by initial thermal decomposition shortly after the melting phase (around 255 °C). TGA results corroborate these observations. The second heating scan indicates a *T*_g_ of 193 °C, indicating the transition from a glassy to an elastomeric state.^29,31^

The DSC curves for the monoliths (Figure 3d) display similar patterns, yet notable differences emerge. The water desorption enthalpy is considerably higher for the monoliths, rising from 19 J g^–1^ for the unprocessed CA to 115 J g^–1^ for the monolith, likely due to its greater porosity, which allows water penetration into the monolith’s inner structure and requires more thermal energy to remove the adsorbed water. Melting and degradation events overlap between 200 and 270 °C, with a distinct secondary endothermic peak around 250 °C, which is associated with polymer decomposition. Due to overlapping peaks, the enthalpy values for melting and decomposition were combined. The *T*_g_ of the monolith was measured at 188 °C, which closely matches that of the unprocessed CA. These findings suggest that morphological changes, particularly increased porosity, significantly influence the thermal properties of the monoliths relative to unprocessed CA.

The X-ray diffraction (XRD) patterns of both materials, cellulose acetate (CA) and porous CA monoliths, exhibited profiles characteristic of semicrystalline materials (Figure S3). Two distinct amorphous halos were observed: a van der Waals halo centered at approximately 2θ ≈ 20° and a low van der Waals halo near 2θ ≈ 10°, in addition to a diffraction band corresponding to crystalline regions at around 2θ ≈ 23.5°. The halo at 2θ ≈ 10° is attributed to increased interplanar spacing caused by bulky substituents, such as the acetyl groups in CA.

Subtle changes were also observed in the low van der Waals halo region near 2θ ≈ 10°, where minor peak shifts and improved definition—characterized by reduced peak broadening and lower full width at half-maximum (fwhm)—were detected. These changes are likely associated with the processing method used in monolith fabrication, which induced modifications in morphology and the distribution of interplanar spacing, reflecting the acquired hierarchical porous structure.

#### 3.1.4 Evaluation of Porosity and Morphology of the CA Monoliths

In this study, total porosity was evaluated using the liquid saturation method with *n*-butanol (eq 1). Nitrogen adsorption–desorption measurements were used to characterize the specific surface area (SSA) and pore structure, employing the BET (Brunauer–Emmett–Teller) and BJH (Barrett–Joyner–Halenda) methods.^32,33^ These analyses provided key morphological insights necessary for designing separation and adsorption systems based on hierarchically porous monolithic materials.

The cellulose acetate (CA) monolith synthesized in this study, using DMF as solvent and *n*-octanol as a nonsolvent, showed an SSA of 34.33 m^2^ g^–1^, a total pore volume of 0.1722 cm^3^ g^–1^, and an average pore diameter of 20.07 nm. According to the IUPAC classification, this material falls within the mesoporous range.^34^ The sample also exhibited a high porosity of 90.68 ± 0.66%. Its isotherm (Figure S4) showed a Type IV (H3) profile, indicating the presence of slit-shaped mesopores and capillary condensation in the multilayer regime.

A nonlyophilized version of the same CA/DMF/octanol monolith presented a significantly lower BET surface area (1.43 m^2^ g^–1^) and a total pore volume of only 0.0124 cm^3^ g^–1^, while maintaining a mesoporous character with an average pore diameter of 34.59 nm and overall porosity of 80.33%. These differences underscore the impact of postsynthesis processing conditions, particularly freeze-drying, in preserving and enhancing pore accessibility.

For comparison, previously reported monoliths based on CA and other solvent/nonsolvent systems exhibit a wide range of surface and porosity characteristics. For example, Zhang et al. (2020) reported a CA/DMF/ethanol monolith with an SSA of 54.45 m^2^ g^–1^, a broad pore-size distribution from 2 to 136 nm, and a high porosity of 92.1% as measured by mercury intrusion. This sample displayed a Type IV isotherm, similar to our freeze-dried sample in terms of porosity type, but with a more even pore distribution.^35^ Conversely, Xin et al. (2017)^8^ described a CA/DMF/hexanol system with an SSA of 41.3 m^2^/g and pore diameters ranging from 40 to 80 nm. Their results exhibited Type V isotherms with H1 hysteresis loops, which typically suggest ink-bottle-shaped pores.^8^

In contrast, a cross-linked CA/acetone/PMDA monolith developed by Tripathi et al. (2017)^36^ displayed much lower textural properties, with an SSA of just 3.4 m^2^ g^–1^ and a pore size range of 2–45 nm. These values reflect the influence of cross-linking chemistry and solvent choice on the resulting porosity and surface characteristics.^36^

Collectively, these results highlight the importance of selecting the appropriate solvent/nonsolvent pair and implementing postprocessing treatments to modulate the hierarchical porosity of CA-based monoliths. The use of octanol in the present study, as a novel nonsolvent in the thermally induced phase separation process, proved effective in producing materials with high porosity and accessible mesopores, particularly when combined with freeze-drying to preserve the porous network. The relatively low skeletal density of 103.12 mg cm^–3^ further supports the presence of a predominantly macroporous structure alongside the mesopores, which is beneficial for fluid transport and rapid adsorption kinetics in environmental remediation applications.^37^

The observed bicontinuous morphology and uniform pore-size transition suggest that the system operated within a metastable region of the DMF/*n*-octanol/CA ternary phase diagram, where phase separation proceeds predominantly via spinodal decomposition. The moderate solvent–nonsolvent miscibility of DMF and *n*-octanol likely shifts the binodal boundary, positioning the working composition near the spinodal region and thus promoting hierarchical pore formation.^11,38^

Scanning electron microscopy (SEM) images of the surface and cross sections were obtained to assess the morphology of the monolithic structures produced, as shown in Figure 4.

**Figure 4:**
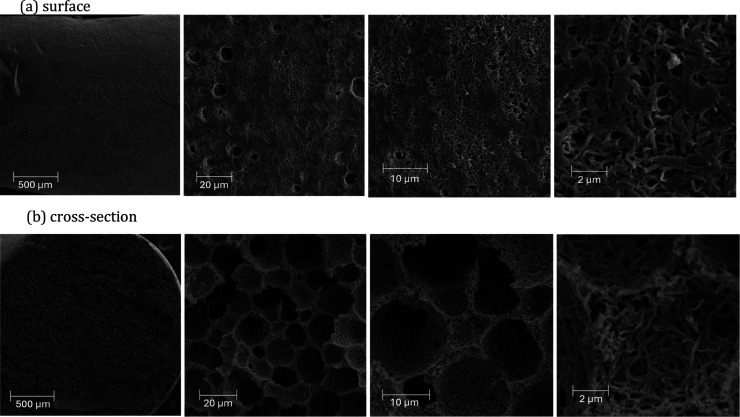
SEM images of the CA monolith showing (a) surface and (b) cross-section views at magnifications of 100×, 2.0 kx, 5.0 kx, and 20.0 kx, revealing the hierarchically porous morphology.

The CA monoliths (Figure 4a) exhibit visually pronounced pores and cavities in their external structure, ranging from less than 0.2 μm to around 10 μm. Its internal morphology is notably distinct, with the formation of a continuous porous structure (Figure 4b), in which regions of lower density and well-defined pores are visible. The CA monolith exhibits a honeycomb-like structure, with well-separated units that reveal the intricate entanglement of the polymeric network.

Additionally, the cross-sectional image shows macropores ranging from 1 to 25 μm, highlighting the hierarchical porosity of these materials.

### 3.2 Adsorption Kinetics and Equilibrium Studies of Crystal Violet on CA Monoliths

#### 3.2.1 Adsorption Kinetics

The adsorption of crystal violet (CV) onto CA monoliths was investigated over a contact time ranging from 1 to 180 min, with initial CV concentrations varying from 0.5 to 5.6 mg L^–1^. As exhibited in Figure 5, the dye removal efficiency (*R* %) depends on both the initial CV concentration and the contact time, reaching approximately 90% removal after 180 min, a period at which the system approaches or reaches adsorption equilibrium.

**Figure 5:**
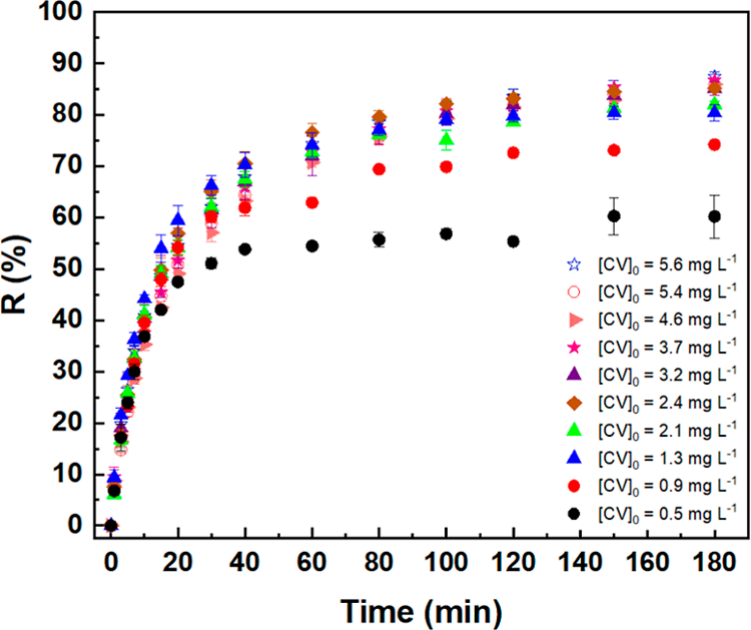
Effect of contact time on the removal efficiency (*R* %) of crystal violet (CV) by CA monoliths. Adsorption was carried out with 10 mL of CV solution (0.5–5.6 mg L^–1^) at pH 6.0 and 22 °C under constant stirring, using 0.070–0.093 g considering the dry mass of CA monoliths.

The removal rate within the first 10 min was consistently higher across all concentrations, due to the large surface area initially available (34.33 m^2^ g^–1^ from ASAP measurements). Nearly 50% of the dye is adsorbed across all concentration curves within the first 25 min. This rapid initial uptake is attributed to the abundance of accessible adsorption sites on the monolith surface. As these sites become increasingly occupied over time, the adsorption rate declines, particularly at higher dye concentrations. As the initial CV concentration increases from 0.5 to 5.6 mg L^–1^, the dye uptake capacity rises from 60% to 87%, indicating that dye concentration plays a significant role in adsorption performance.

Dye molecules may migrate from the outer surfaces into the adsorbent’s internal regions, leading to the accumulation of aggregates at active sites.^39^ A higher concentration gradient increases the driving force required to overcome mass-transfer resistances between the aqueous and solid phases, particularly within the mesoporous regions. Additionally, interconnected macropores or voids facilitate further transport within the monolith, thereby increasing the equilibrium adsorption capacity until the adsorbent reaches saturation.^40^ This phenomenon is illustrated in Figure S5, where the saturation of active sites progressively advances, achieving approximately 86% dye removal and an adsorption capacity of 0.6 mg g^–1^ at higher concentrations.

In more diluted systems, CV molecules experience prominent solvation layers, which may hinder adsorption onto the hydrophobic cellulose acetate monolith surface. This observation is supported by the equilibrium time, which is reached after 60 min for the 0.5 mg L^–1^ solution and after 120 min for all other concentrations (Figure 5). Although equilibrium was achieved more quickly at the lowest concentration, its overall adsorption capacity was comparatively lower.

Several kinetic models are commonly used to describe adsorption rates and mechanisms in aqueous systems. While the adsorption rate is essential for evaluating an adsorbent’s efficiency, the mechanism helps differentiate between physisorption and chemisorption. To better understand the governing mechanism, the experimentally determined equilibrium adsorption capacity (*q*_eq_) was fitted to pseudo-first-order, pseudo-second-order, Elovich, and intraparticle diffusion models over the studied CV concentration range (0.5–5.6 mg L^–1^), as shown in Figure 6a. The kinetic data obtained for these models are displayed in Table S2.

**Figure 6:**
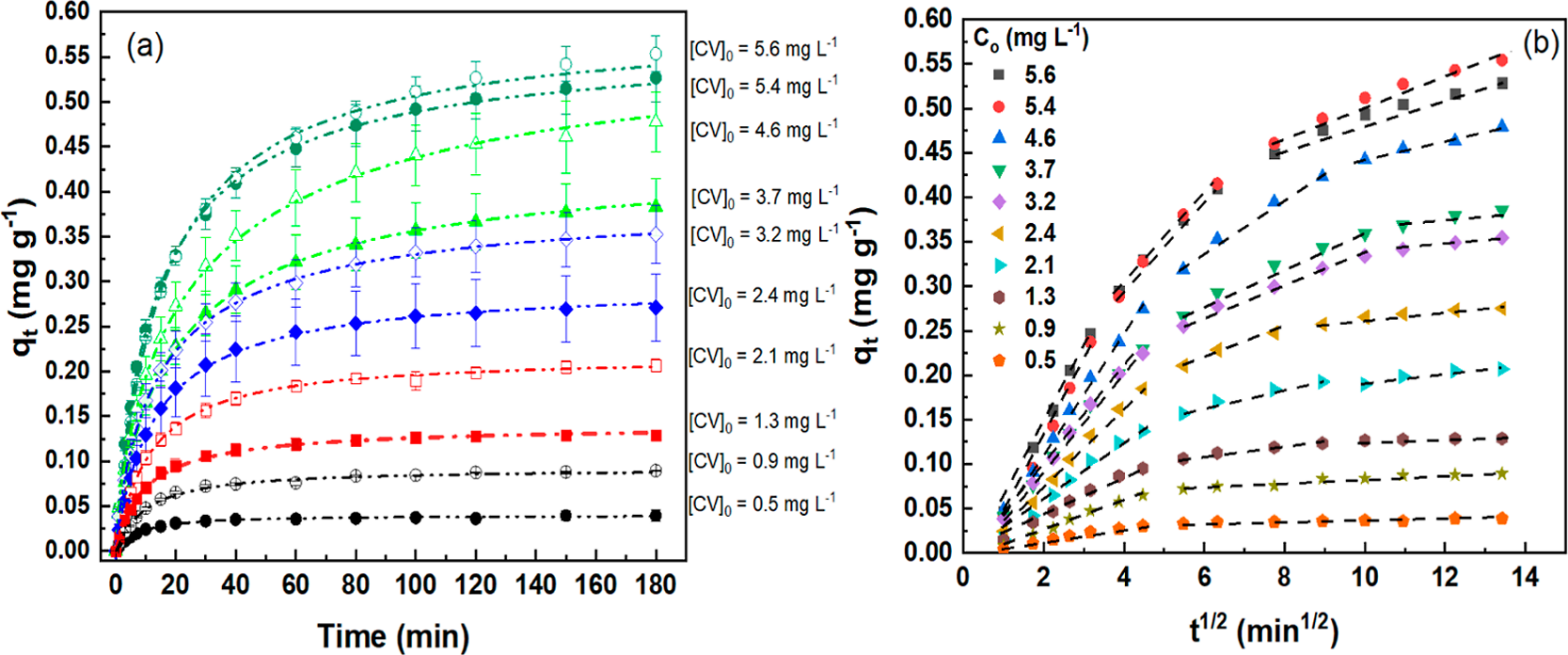
(a) Amount of CV adsorbed (*q*_*t*_) by CA monoliths after 180 min at different initial concentrations (0.5–5.6 mg L^–1^); (b) intraparticle diffusion model (*q*_*t*_ vs *t* ^1/2^), showing multilinear behavior typical of the external diffusion, intraparticle diffusion, and equilibrium stages. Adsorption was performed with 10 mL of CV solution (0.5–5.6 mg L^–1^) at pH 6.0 and 22 °C under constant stirring.

Nonlinear fitting was evaluated using statistical criteria, including the coefficient of determination (*R* ^2^), the adjusted *R* ^2^, the sum of squared errors (SSE), and the root-mean-square error (RMSE). Parameter estimation and model selection were conducted in MATLAB, with the results summarized in Table S2.

The results demonstrated that at lower concentrations (0.5, 0.9, 1.3, and 2.1 mg L^–1^), the pseudo-second-order model provided the best fit, with *R* ^2^ values of 0.9952, 0.9963, 0.9990, and 0.9990, respectively. At higher concentrations (above 2.4 mg L^–1^), both the pseudo-second-order and Elovich models exhibited high *R* ^2^ values (above 0.99). Thus, *R* ^2^ alone was insufficient to identify the best model, necessitating the use of additional criteria, such as RMSE. Using this criterion, the pseudo-second-order model remained the most appropriate, as the predicted *q*_eq_ values were close to the experimental data, with an RMSE of 1.09 × 10^–5^. Furthermore, the equilibrium adsorption capacities predicted by this model aligned closely with the experimental values (Table S2).

Similar adsorption behavior has been reported for cationic dye adsorption onto various biomass-based adsorbents.^41–43^ These findings suggest that electrostatic interactions between the dye and the polymer surface play a significant role in adsorption, following a chemisorption mechanism described by the pseudo-second-order model.^44^ Understanding the role of electrostatic forces requires knowledge of the chemical structures of both the dye and the polymer. Cellulose acetate (CA) contains functional groups, such as carbonyl and hydroxyl groups, which can interact with dye molecules. CA exhibits a surface charge of −35.7 at pHs above 5, enabling strong electrostatic interactions with cationic dyes, such as CV, which are likely to contribute to chemisorption, as evidenced by the pseudo-second-order model fit.^45,46^

Although the Elovich model also showed a reasonable fit across the entire concentration range, its *R* ^2^ values were generally lower than those of the pseudo-second-order model. The Elovich model typically describes chemisorption kinetics and may also reflect heterogeneous surface interactions. Therefore, its fit suggests that the adsorption process may involve cooperative chemisorption mechanisms.

When evaluated using the intraparticle diffusion model (Figure 6b), the *q*_t_ vs *t* ^1/2^ plot exhibited more than one linear region, indicating that multiple processes influence adsorption, with three distinct stages observed: an initial boundary layer diffusion stage characterized by rapid external surface adsorption, followed by a gradual adsorption stage where intraparticle diffusion is rate-limiting, and finally, a plateau region indicating equilibrium, where diffusion slows due to lower solute concentration.^22,40,45^ These observations suggest that multiple mechanisms may simultaneously control adsorption.

#### 3.2.2 Adsorption Isotherms and Capacity

At equilibrium (180 min of contact time), the adsorption capacity (*q*_e_) was assessed for various initial CV concentrations, ranging from 0.5 to 50 mg L^–1^ (Figure S6). The maximum adsorption capacity observed at an initial concentration of 50 mg L^–1^ was 0.82 mg g^–1^. Although this value is modest relative to lignocellulosic-based adsorbents, two critical factors should be acknowledged: (i) the Brunauer–Emmett–Teller (BET) specific surface area, which indicates that the material is mesoporous based on pore diameter, and (ii) the inherent properties of the adsorbent. The monoliths used in this study exhibited a BET surface area of 34.33 m^2^ g^–1^ and an average pore diameter of 20.07 nm, characterizing them as mesoporous. Despite their high porosity (90.68 ± 0.66%), SEM micrographs indicate that this porosity predominantly comprises macropores and large voids, which facilitate mass transport but may not significantly contribute to adsorption. Furthermore, CA is more hydrophobic than other lignocellulosic materials, which may weaken its interactions with dye molecules compared to those of cellulose-based fibers. In this sense, it is essential to emphasize that the monoliths produced can be modulated with respect to both their hierarchically porous morphological structure and the presence of functional groups, thereby improving the adsorption of dyes or other substances of interest.

The Langmuir and the Freundlich isotherm models were applied to the experimental data (Figure 7) to better understand the adsorption mechanism.

**Figure 7:**
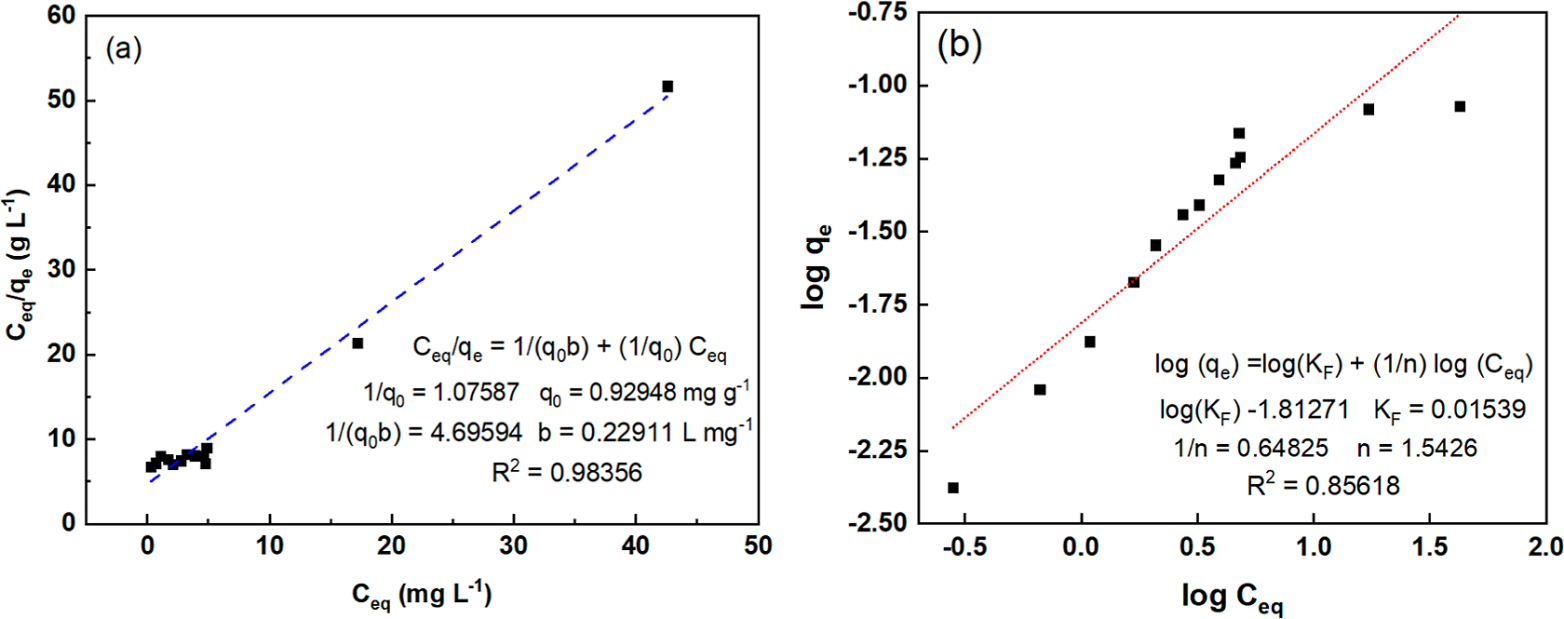
Adsorption isotherms of CV on CA monoliths fitted to the linearized (a) Langmuir and (b) Freundlich models. Experiments were conducted at 22 °C and pH 6.0, using 10 mL of CV solution (0.5–5.6 mg L^–1^).

The Langmuir model assumes monolayer adsorption on a homogeneous surface, whereas the Freundlich model accounts for multilayer adsorption on a heterogeneous surface. The results in Figure 7 indicate that adsorption is best described by the Langmuir model, suggesting that adsorption occurs primarily as a monolayer process. The maximum adsorption capacity (*q*_0_) obtained from the Langmuir model was 0.92948 mg g^–1^. Although the Freundlich 1/*n* value falls within the favorable adsorption range (0.1–0.5), the coefficient of determination for the Freundlich model was lower than that for the Langmuir model, indicating a weaker fit.

The Langmuir separation factor (*R*_L_), calculated using eq 7, further supports the model’s applicability.

7 $$R_{L}=\frac{1}{1+b\times C_{0}}$$

where *b* is the Langmuir constant, and *C*_o_ is the initial concentration of the dye.

As shown in Figure S7, *R*_L_ values range between 0 and 1 for all studied concentrations (0.5–50 mg L^–1^), confirming that the adsorption process is favorable. Decreasing *R*_L_ values indicate that adsorption becomes more favorable at higher dye concentrations.

#### 3.2.3 Desorption Studies

From an economic and application perspective, the ability to desorb the adsorbed dye is crucial for both monolith reuse and dye recovery. Different extractant solutions, including buffered aqueous solutions at pH 2 and 9, as well as ethanol and methanol, were tested to evaluate desorption efficiency. Figure S8 shows the visual progression of CV desorption in methanol, while Figure 8 summarizes the desorption efficiency for each solvent.

**Figure 8:**
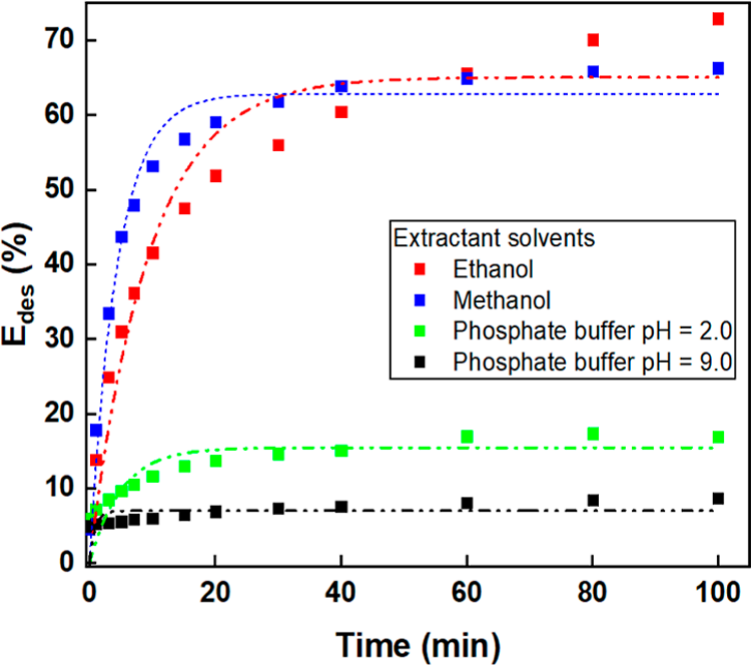
Desorption efficiency (%) of CV after 180 min of contact with CA monoliths, using different extracting solvents: methanol, ethanol, and buffered aqueous solutions at pH 2 and 9.

Desorption percentages were calculated using eqs 5 and 6. The highest desorption efficiencies (∼70%) were achieved using ethanol and methanol, whereas pH adjustments in aqueous solutions did not significantly enhance dye removal. These findings suggest that the choice of extractant solution is crucial, as it must overcome the forces that bind the dye to the polymer structure.

Moreover, desorption appears to be intensely dependent on temperature, desorption time, eluent volume, and the chemical properties of the solvent. Although the present work did not systematically aim to optimize these parameters for dye removal from the monolith, the results suggest that complete desorption of CV could be achieved under more favorable conditions, such as higher temperatures, longer contact times, and larger eluent volumes. Still, even without such optimization, the ∼70% recovery observed highlights the excellent regeneration potential of the monoliths. Similar or even lower desorption efficiencies have been reported in the literature.^47–50^

The results demonstrate that CA-based monoliths can be tailored for various applications by modulating their hierarchical porosity and functional groups, thereby enhancing adsorption efficiency for dyes and other target substances.

## 4 Conclusions

This study establishes a sustainable and effective route to fabricate hierarchically porous cellulose acetate (CA) monoliths via the TIPS/NIPS method, employing *n*-octanol as the key nonsolvent. The deliberate selection of *n*-octanol, owing to its low volatility and controlled miscibility, enabled a finely tuned phase separation process, leading to the formation of a well-defined, bicontinuous porous network. The resulting monoliths exhibit high porosity (90.68%), a mesoporous character (BET surface area of 34.33 m^2^ g^–1^), and a sponge-like interconnected morphology.

This hierarchical structure is not merely morphological but fundamental to performance: macropores facilitate efficient mass transport, while mesopores provide extensive surface area for adsorption. Adsorption experiments using crystal violet (CV) confirmed a chemisorption-driven process consistent with the pseudo-second-order kinetic model and Langmuir isotherm, indicating monolayer adsorption on a homogeneous surface. Electrostatic attraction between the cationic dye and the negatively charged CA surface is the primary interaction mechanism.

While the maximum adsorption capacity (0.929 mg g^–1^) is relatively modest compared with conventional adsorbents, it is consistent with values typically reported for unmodified cellulose acetate. Importantly, these monoliths offer practical advantages: they are single-piece, mechanically stable materials that eliminate postfiltration steps and are compatible with flow-through or column systems. Furthermore, the efficient desorption of CV using ethanol and methanol (up to 70%) demonstrates their potential for reuse, reinforcing their economic and environmental viability.

The integration of *n*-octanol into the TIPS/NIPS process provides a novel and scalable pathway to engineer CA-based monoliths with tunable hierarchical porosity, with strong promise for sustainable adsorption, wastewater remediation, and other advanced separation technologies.

## Supplementary Material



## Data Availability

The data underlying this study are available in the published article and its Supporting Information.

## Notes

Characterization data includes UV–vis and FTIR spectra, X-ray diffraction curves, and nitrogen adsorption/desorption isotherms. Thermal data from the TGA curves and kinetic data for the evaluated models are available in Tables S1 and S2, respectively. It also features graphs showing the effects of the initial CV concentration on dye adsorption, adsorption capacity, and the Langmuir separation factor. Finally, photographs of the dye desorption progress from the monoliths are exhibited (PDF)
