# Supplementary material for: Processing Cellulose Acetate into Hierarchically Porous Monoliths via Thermally Impacted Nonsolvent-induced Phase Separation with Octanol: Application in Dye Adsorption
Source: ACS Omega. 2025 Dec 22;11(1):1323–34. doi: 10.1021/acsomega.5c08761 (PMC12809551; doi:10.1021/acsomega.5c08761)
Supplement: Supplementary file 1 [file ao5c08761_si_001.pdf]

Processing Cellulose Acetate into Hierarchically Porous Monoliths via Thermally Impacted Non-Solvent-Induced Phase Separation with Octanol: Application in Dye Adsorption

Poliana Ricci[a], Brenda F. Silva[a], Marcos V. Ferreira[a], Henrique A. Sobreira[a],[c], Allyson L. R. Santos[a], Anizio M. Faria[a,b], Rosana M. N. Assunção[a,b*].

[a] *Institute of Chemistry, Federal University of Uberlândia, Uberlândia, 38400-902, Brazil.*

[b] *Institute of Exact and Natural Sciences of Pontal, Federal University of Uberlândia, Ituiutaba, 38304-402, Brazil.*

[c] *Federal Institute of Education, Science and Technology of Triângulo Mineiro, Uberaba, 38305-200, Brazil*

* Corresponding author: rosana.assuncao@ufu.br

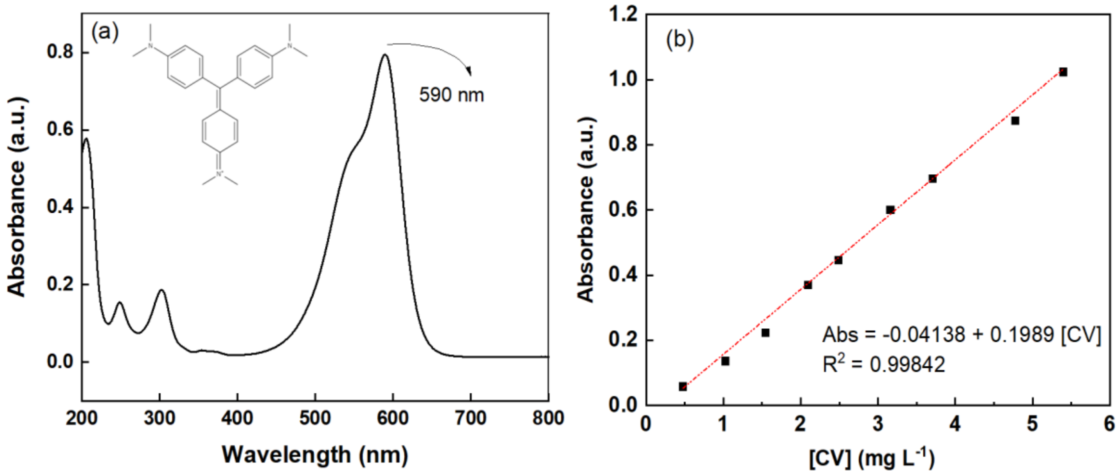

Figure S1. (a) UV-vis spectrum for the CV stock solution; (b) standard curve for CV concentration ranging from 0.5 to 5.6 mg L^{-1} used for CV quantification on the adsorption/desorption tests. Insert (a) chemical structure of CV.

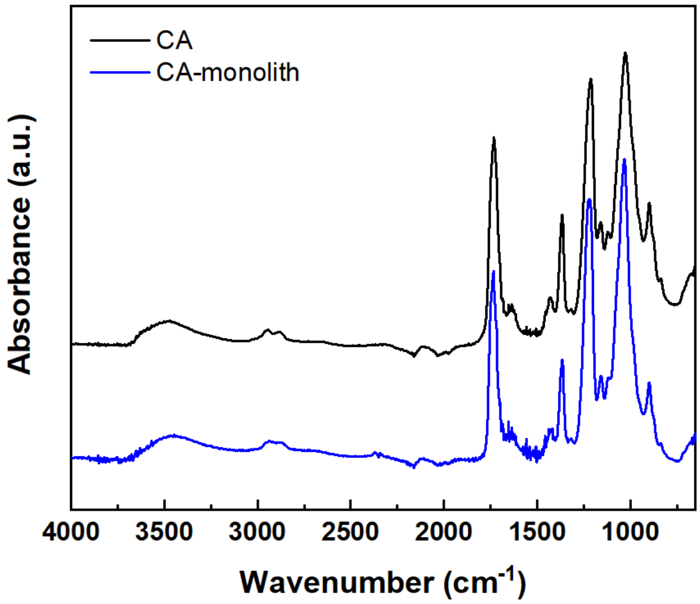

Figure S2. FTIR spectra of the unprocessed CA and the CA/DMF/octanol monoliths.

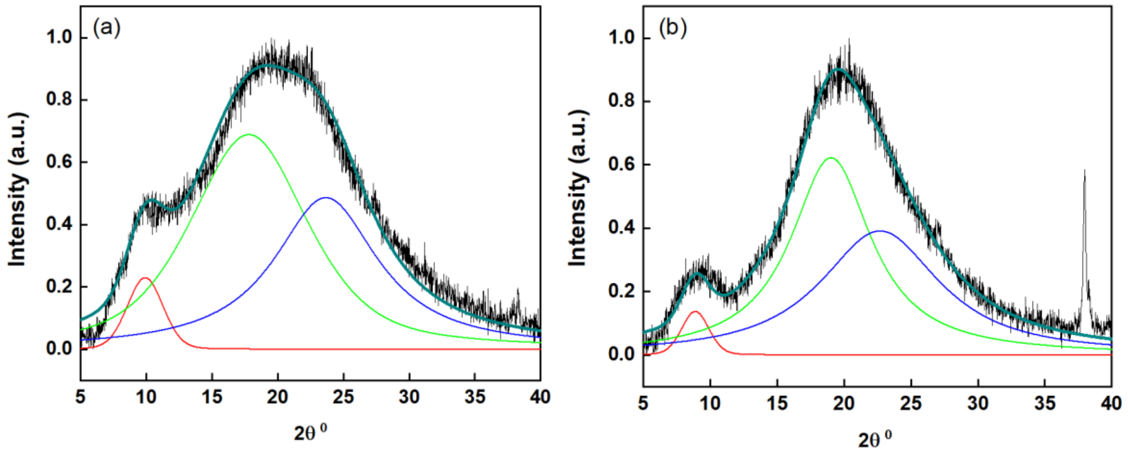

Figure S3. X-ray diffraction curves for (a) CA and (b) CA monolith. The blue line represents the deconvolution of the crystalline peak, while the red and green lines are attributed to the two amorphous halos.

Both CA and CA monolith samples displayed characteristic profiles of semicrystalline materials, followed by low crystallinity indices. Two distinct halos were observed: the first, corresponding to the van der Waals halo, appears at 2θ around 20°, while the second, referred to as the low van der Waals halo, is seen at 2θ close to 10°. These features indicate the presence of amorphous or semicrystalline structures. They are attributed to the

increased interplanar spacing resulting from bulky lateral groups, such as the acetyl groups present in CA.

Slight differences were observed between the X-ray diffraction (XRD) patterns of cellulose acetate (CA) and the monoliths, particularly in the peak at approximately 2θ = 10°, which showed minor shifts and broadening. These alterations are likely attributable to the porous morphology developed during monolith synthesis. A characteristic crystalline peak for acetates was also detected at around 2θ = 23.5°.

The deconvolution of the diffraction peaks facilitated the estimation of crystallinity indices (see colored lines in Fig. S3). The XRD profiles indicated a slight peak narrowing in the monoliths compared to CA, resulting in crystallinity indices of 45.11% for CA and 46.63% for the monoliths, thereby confirming the low crystallinity of all samples.

Therefore, the monoliths constructed from low-crystallinity cellulose acetate (CA) showed no significant change in crystallinity during development. Instead, the primary factor influencing their properties is the porous structure's surface area.

Table S1. T_{onset}, T_{endset}, and T_{max} (°C) related to the main thermal events presented in the TGA/DTG curves for CA and CA monolith.

**Sample**	**1st event**	**Additional event***	**2nd event**				**residue at 600 °C**
	**% mass loss**	**T_{max} (°C)**	**T_{onset} (°C)**	**% mass loss**	**T_{endset} (°C)**	**T_{max} (°C)**	**(%)**
CA	3.9	----	335.0	82.9	377.5	358.0	9.8
CA monolith	1.4	236.6	315.7	74.3	369.6	352.0	9.6

* Thermal event attributed to the morphological changes promoted by the monolith’s porous structure.

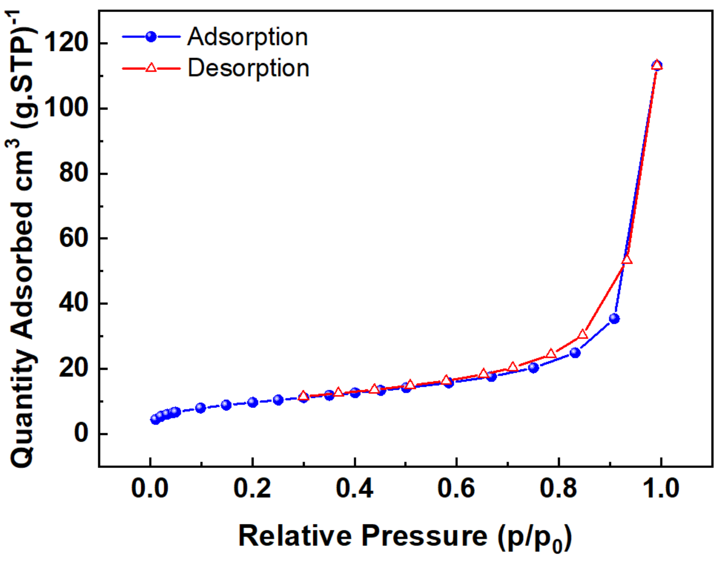

Figure S4. Nitrogen adsorption/desorption isotherms for the CA monolith.

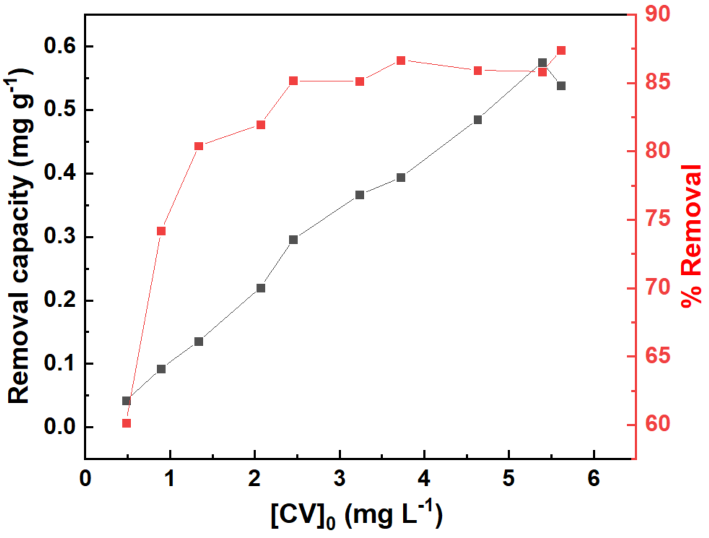

Figure S5. Effect of CV initial concentration on the dye adsorption.

Table S2. Kinetic data for the pseudo-first-order, pseudo-second-order, Elovich, and intraparticle diffusion models.

**Kinetic models and their parameters**	**Concentration (mg L^{-1})**									
	**0.4795**	**0.8943**	**1.3386**	**2.0665**	**2.4521**	**3.2369**	**3.7248**	**4.6279**	**5.3903**	**5.6124**
Q_{eq} (mg g^{-1}) (Experimental)	0.0042	0.0091	0.0133	0.0214	0.0296	0.0365	0.0393	0.0483	0.0542	0.5703
**Pseudo-first-order**										
Q_e (mg g^{-1})	0.0038	0.0086	0.013	0.0201	0.0272	0.0341	0.0372	0.0459	0.0532	0.0506
K_1 (min^{-1})	0.1012	0.0786	0.0828	0.0679	0.0641	0.0624	0.0541	0.0502	0.0524	0.0619
R^2	0.9892	0.9863	0.9866	0.9866	0.9905	0.9772	0.9791	0.9785	0.9832	0.9766
R^2adj	0.9884	0.9853	0.9857	0.9857	0.9898	0.9756	0.9776	0.9770	0.9821	0.9750
SSE	2.77×10^{-7}	1.98×10^{-6}	1.05×10^{-5}	1.05×10^{-5}	1.38×10^{-5}	5.04×10^{-5}	5.57×10^{-5}	8.80×10^{-5}	9.49×10^{-5}	1.15×10^{-4}
RMSE	1.41×10^{-4}	3.76×10^{-4}	8.68×10^{-4}	8.68×10^{-4}	9.92×10^{-4}	1.90×10^{-3}	1.99×10^{-3}	2.51×10^{-3}	2.60×10^{-3}	2.86×10^{-3}
**Pseudo-second-order**										
Q_e (mg g^{-1})	0.0041	0.0096	0.0142	0.0226	0.0307	0.0386	0.0425	0.0529	0.0613	0.0573
K_2 (g mg^{-1} min^{-1})	33.65	10.27	7.736	3.787	2.618	2.041	1.558	1.142	1.019	1.357
R^2	0.9952	0.9963	0.9990	0.9990	0.9997	0.9978	0.9976	0.9969	0.9990	0.9979
R^2adj	0.9949	0.9961	0.9989	0.9989	0.9997	0.9977	0.9974	0.9966	0.9989	0.9977

SSE	1.23×10^{-7}	5.27×10^{-7}	7.92×10^{-7}	7.92×10^{-7}	3.69×10^{-7}	4.78×10^{-6}	6.50×10^{-6}	1.28×10^{-5}	5.86×10^{-6}	1.04×10^{-5}
RMSE	9.37×10^{-5}	1.94×10^{-4}	2.38×10^{-4}	2.38×10^{-4}	1.62×10^{-4}	5.85×10^{-4}	6.82×10^{-4}	9.56×10^{-4}	6.47×10^{-4}	8.63×10^{-4}
**Elovich**										
a (mg g^{-1} min^{-1})	0.0016	0.0022	0.0039	0.0040	0.0050	0.0063	0.0054	0.0059	0.0070	0.0092
b (g mg^{-1})	1399	543.1	384.6	223.0	162.3	129.4	112.5	88.42	75.98	86.71
R^2	0.9572	0.9723	0.9811	0.9811	0.9806	0.9918	0.9940	0.9951	0.9926	0.9927
R^2adj	0.9541	0.9703	0.9797	0.9797	0.9793	0.9912	0.9935	0.9954	0.9921	0.9922
SSE	1.10×10^{-6}	4.00×10^{-6}	1.49×10^{-5}	1.49×10^{-5}	2.80×10^{-5}	1.81×10^{-5}	1.60×10^{-5}	1.88×10^{-5}	4.20×10^{-5}	3.58×10^{-5}
RMSE	2.80×10^{-4}	5.34×10^{-4}	2.38×10^{-4}	2.38×10^{-4}	1.41×10^{-3}	1.14×10^{-3}	1.07×10^{-3}	1.16×10^{-3}	1.73×10^{-3}	1.60×10^{-3}
**Intraparticle diffusion**										
c (mg g^{-1})	0.0011	0.0020	0.0032	0.0040	0.0052	0.0065	0.0059	0.0066	0.0076	0.0094
K (mg g^{-1} $min^{-0.5}$)	0.0003	0.0007	0.0010	0.0016	0.0022	0.0027	0.0030	0.0038	0.004	0.0041
R^2	0.7660	0.8281	0.8152	0.8565	0.8607	0.8897	0.9109	0.9226	0.9100	0.8913
R^2adj	0.7493	0.8158	0.8021	0.8463	0.8508	0.8818	0.9046	0.9171	0.9036	0.8835
SSE	6.00×10^{-6}	2.48×10^{-5}	5.57×10^{-5}	1.13×10^{-4}	2.01×10^{-4}	2.44×10^{-4}	2.37×10^{-4}	3.17×10^{-4}	5.09×10^{-4}	5.34×10^{-4}
RMSE	6.55×10^{-4}	1.33×10^{-3}	2.00×10^{-3}	2.84×10^{-3}	3.79×10^{-3}	4.18×10^{-3}	4.11×10^{-3}	4.76×10^{-3}	6.03×10^{-3}	6.17×10^{-3}

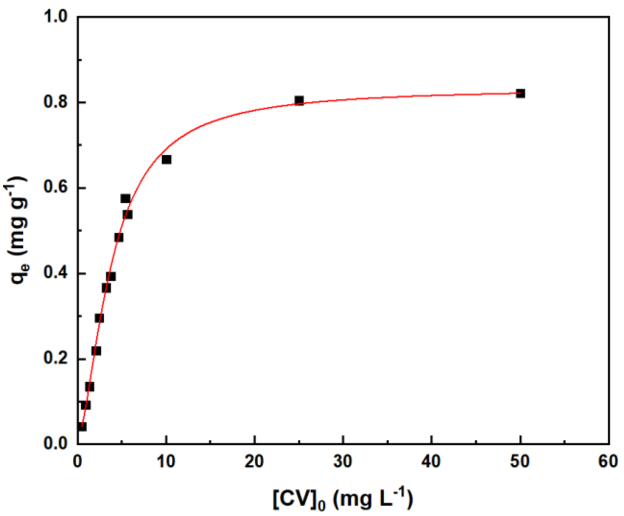

Figure S6. Adsorption capacity (q_e) as a function of the initial concentration of CV, ranging from 0.5 to 50 mg L^{-1}, T = 22 °C.

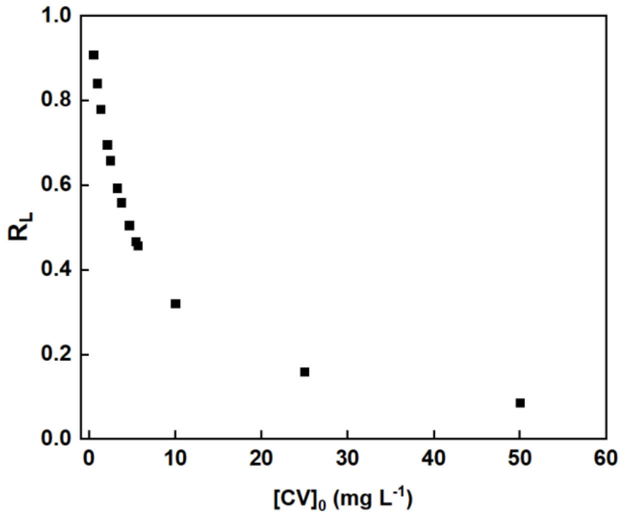

Figure S7. Langmuir separation factor (R_L) as a function of the initial concentration of CV, ranging from 0.5 to 50 mg L^{-1}, T = 22 °C.

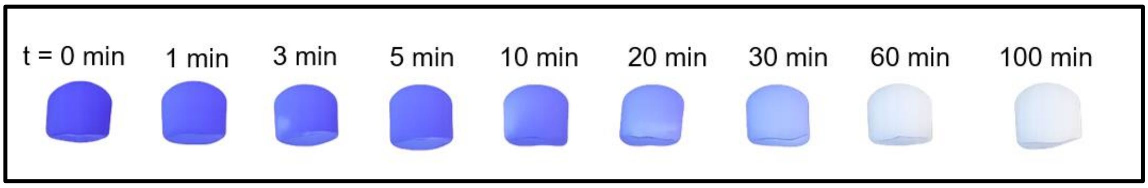

Figure S8. Photographs of the CV desorption progress from the CA monoliths using methanol as the extractant solution.
